# Comparison of false-discovery rate for genome-wide and fine mapping regions

**DOI:** 10.1186/1753-6561-1-s1-s148

**Published:** 2007-12-18

**Authors:** Meredith E Tabangin, Jessica G Woo, Chunyan Liu, Todd G Nick, Lisa J Martin

**Affiliations:** 1 Department of Pediatrics, Cincinnati Children's Hospital Medical Center, 3333 Burnet Avenue, MLC 5041, Cincinnati, Ohio 45229-3039, USA; 2 University of Cincinnati School of Medicine, 231 Albert Sabin Way, Cincinnati, Ohio 45267, USA

## Abstract

With technological advances in high-throughput genotyping, it is not unusual to perform hundreds of thousands of tests for each phenotype. Thus, correction to control type I error is essential. The false-discovery rate (FDR) has been successfully used in genome-wide expression data. However, its performance has not been evaluated for association analysis. Our objective was to analyze the Genetic Analysis Workshop 15 simulated data set, with answers, to evaluate FDR for genome-wide association and fine mapping. In genome-wide analysis, FDR performed well, with good localization of positive results. However, in fine mapping, all tested methods performed poorly, producing a high proportion of significant results. Thus, caution should be used when employing FDR for fine mapping.

## Background

Multiple testing within research studies takes many forms, including multiple comparisons regarding a single outcome, multiple outcomes, or multiple secondary analyses. Each scenario increases the overall probability of false-positive outcomes. With technological advances in high-throughput genotyping, it is not unusual to perform hundreds of thousands of tests for each phenotype in genome-wide association studies. Thus, strategies are required to control type I error rate.

The false-discovery rate (FDR) was originally proposed by Benjamini and Hochberg as a method to adjust for multiple comparisons [1]. Less conservative than traditional control of family-wise error (FWE) rate (likelihood of making at least one type I error over all tests), FDR is the expected proportion of 'significant' tests that are truly null. FDR was extended by Storey with the *q*-value, the FDR analogue of the *p*-value [2]. The *q*-value is the proportion of significant single-nucleotide polymorphisms (SNPs) that will be false positives for a given threshold. For example, if we want to review SNPs with *q*-values ≤ 0.05 and this yields 200 SNPs, then approximately 10 of these 'significant' SNPs are expected to be null. Storey assumes that null *p*-values are uniformly distributed, and can thus be used to determine the *q*-value. These assumptions are valid for genome-wide expression data [2]. However, performance of FDR in genome-wide association studies has not been evaluated with simulated data.

In genome-wide linkage and association studies, it is common practice to follow up previously identified linkage and association signals with dense genotyping in that area (e.g., fine mapping). Genotyping a large number of SNPs in a region may introduce non-independence due to linkage disequilibrium (LD). Additionally, we would expect an increase in the number of true positives, as these regions were selected because of previous evidence of linkage or association. Unfortunately, the impact of non-independence and increased true positives on the distribution of null *p*-values and FDR performance has not been explored. Therefore, our objective was to analyze the Genetic Analysis Workshop 15 (GAW15) simulated data set, with answers, to evaluate FDR for genome-wide association and fine mapping.

## Methods

### Data

GAW15 simulated rheumatoid arthritis (RA) data consists of 1500 families with an affected sibling pair and 2000 unaffected controls. The authors had knowledge of the simulated answers at the time of analyses.

All 9187 SNPs on 22 autosomes were analyzed in 11 replicates (90–100). The dense map of 17,820 SNPs on chromosome 6 was used to further model an analysis of follow-up of a known linkage peak region. Of these SNPs, 2094 within 10 cM flanking the causative allele at locus DR, as provided in the answers, were selected for analysis.

### Statistical analysis

CCREL in the computer program R [3] was used for SNP association analysis. This package permits case-control analysis controlling for familial relationships. Because the GAW15 data provided information on family trios, as well as controls, this was an optimal method for analysis. *p*-values for each replicate were imported into QVALUE and analyzed using default settings and a FDR of 0.05 [2]. To compare FDR performance in genome-wide versus fine mapping, we compared the number of tests meeting this FDR threshold for each replicate and the average estimated π_0 _(the probability that a given hypothesis is truly null). To compare methods, FDR was also estimated using the method of Benjamini and Hochberg [1] and a robust method assuming discrete *p*-values [4]. Results from FDR were compared with a nominal *p*-value threshold of 0.05 and Bonferroni corrected thresholds of 0.05/9187 = 5*10^-6 ^for genome-wide and 0.05/2094 = 2*10^-5 ^for fine mapping.

To examine the underlying assumption of a uniform distribution of *p*-values, we inspected the *p*-value distribution from all replicates combined. To explore the empirical false-positive (FP) rate, SNPs identified as significant in the genome-wide association analysis were examined for proximity to any of the modeled causative loci. Significant SNPs within either 10 cM or 5 cM of causative loci were labeled 'true positives' (TP), and the proportion of all significant SNPs labeled 'true positive' and 'false positive' (where FP = 1 - TP) was calculated.

Because non-independence could also influence the results, we examined LD between the SNP at the DR locus and 186 SNPs flanking DR. LD was calculated using *r*^2^. Spearman's rank correlation between LD and *p*-values for the association was used to determine if the *p*-values were related to LD.

## Results

### Genome-wide association

**Figure 1:**
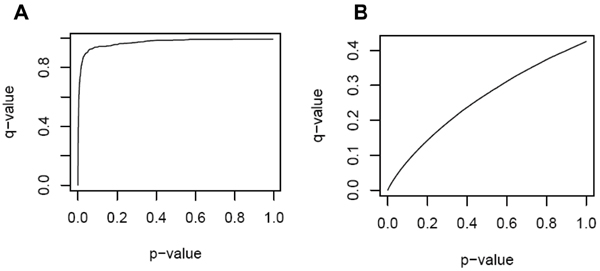
***p*-Values versus *q*-values for all replicates combined**. A, Genome-wide; B, fine mapped region.

**Figure 2:**
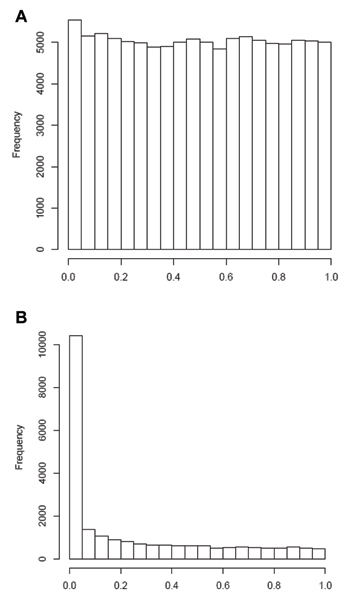
**Histogram of *p*-values for all replicates combined**. A, Genome-wide (*n *= 9187 SNPs per replicate); B, fine mapped region (*n *= 2094 SNPs per replicate).

An average of 5.5% of SNPs was significant at *p *≤ 0.05 compared to 0.27% and 0.21% of SNPs using FDR and Bonferroni adjustment, respectively. Estimated π_0 _averaged 0.99 (Table 1). The plot of *q*-values against the corresponding *p*-values exhibited exponential increases in *q*-values associated with small increases in *p*-values close to 0 (Fig. 1A). The *p*-values appeared uniformly distributed above *p *= 0.10, while the frequency at *p *≤ 0.05 was only slightly higher than baseline (Fig. 2A).

**Table 1:** Results from FDR program QVALUE in 11 replicates

	Genome-wide (*n *= 9187 SNPs)				Fine mapping (*n *= 2094 SNPs)			
		
	Number significant (%)				Number significant (%)			
				
Replicate	*p *≤ 0.05	Bonferroni	FDR	FDR π_0_	*p *≤ 0.05	Bonferroni	FDR	FDR π_0_
90	498 (5.4)	21 (0.23)	28 (0.30)	0.982	944 (45.1)	489 (23.4)	1001 (47.8)	0.339
91	483 (5.3)	21 (0.23)	28 (0.30)	0.997	948 (45.3)	448 (21.4)	940 (44.9)	0.464
92	484 (5.3)	23 (0.25)	24 (0.26)	1	939 (44.9)	459 (21.9)	1082 (51.7)	0.248
93	501 (5.5)	18 (0.20)	25 (0.27)	1	914 (43.7)	435 (20.8)	910 (43.5)	0.468
94	526 (5.7)	19 (0.21)	23 (0.25)	0.989	899 (43.0)	444 (21.2)	879 (42.0)	0.471
95	493 (5.4)	20 (0.22)	26 (0.28)	0.992	1010 (48.3)	432 (20.6)	1069 (51.1)	0.355
96	494 (5.4)	18 (0.20)	26 (0.28)	1	945 (45.2)	463 (22.1)	966 (46.1)	0.411
97	533 (5.8)	17 (0.19)	22 (0.24)	0.992	1000 (47.8)	443 (21.2)	1010 (48.2)	0.439
98	500 (5.4)	19 (0.21)	22 (0.24)	0.958	939 (44.9)	437 (20.9)	926 (44.2)	0.467
99	501 (5.5)	21 (0.23)	26 (0.28)	1	923 (44.1)	459 (21.9)	915 (43.7)	0.486
100	528 (5.8)	20 (0.22)	22 (0.24)	0.988	955 (45.6)	428 (20.4)	981 (46.8)	0.366
Average	503.7 (5.5)	19.7 (0.21)	24.7 (0.27)	0.991	946.9 (45.2)	448.8 (21.4)	970.8 (46.4)	0.410

FP rates for significant SNPs differed greatly by method. For the nominal *p *≤ 0.05 threshold, 4920 of 5541 significant SNPs (89%) were >10 cM and 5003 (90%) were >5 cM from a causative locus. Even on chromosomes with a causative locus, the 5-cM FP rate was marginally lower on only three of the six chromosomes (6 [52%], 11 [75%], and 18 [77%]). Using FDR thresholds, only 9 of the 272 SNPs were >5 cM from a causative locus, for a 5-cM FP rate of 3.3%, with the vast majority (96.3%) within 3 cM of a causative locus. Bonferroni thresholds identified 217 significant SNPs, all but one of which (0.46%) was within 3 cM of causative loci. Loci that mediated RA hazard or increased RA severity were not identified using any method.

### Fine mapping

In contrast, using the dense chromosome 6 SNPs, an average of 45.2% of SNPs was significant at *p *≤ 0.05 compared with 46.4% and 21.4% of SNPs using FDR and Bonferroni, respectively. A cluster of 186 significant SNPs spanned 2 Mb around DR, but these accounted for less than 20% of significant associations.

The estimate of π_0 _was much lower, averaging 0.41 (Table 1). The plot of *q*-values against the corresponding *p*-values showed nearly complete correspondence between these measures (Fig. 1B). In addition, while the *p*-values appear uniformly distributed above *p *= 0.10, the frequency at *p *≤ 0.05 is approximately 10-fold higher than baseline (Fig. 2B). Utilization of the Benjamini and Hochberg method did not appreciably change the results (data not shown). However, using a robust FDR method reduced the number of significant results to 581 SNPs (27.7%).

In the SNPs flanking DR, average LD (*r*^2^) was 0.20, with very little correlation between LD and the *p*-value of the association (Spearman's *r *= -0.07, NS).

## Conclusion

Genome-wide FDR reduced the number of results identified as significant compared with unadjusted *p*-value thresholds (e.g., *p *≤ 0.05), and similar to Bonferroni adjusted *p*-value thresholds. Defining false positives as greater than 5 cM from a causal locus, SNPs identified by FDR exhibited only a 3.3% FP rate; thus, FDR was empirically more conservative than expected in this analysis.

For fine-mapped regions, FDR and *p*-value methods all produced a high proportion of significant results. This breakdown in FDR in fine mapping may be due to the extremely skewed distribution of *p*-values seen in the region harboring disease-causing alleles. Pounds and Cheng [4] have noted the importance of the distribution of the *p*-values in FDR estimation. When FDR was estimated using a robust methodology, there was a marked reduction in the number of significant tests; however, a large number of significant results remained. Likewise, the low correlation between pair-wise LD and *p*-values suggests that the large number of significant findings was not due to LD. However, because the causative locus was modeled as a tri-allelic marker, the pairwise LD between bi-allelic SNPs may not capture the true extent of LD with the DR locus.

We suspect that these findings were due to the very strong association (*p *< 10^-300^) between the DR locus (a tri-allelic marker) and RA. Because GAW15 Problem 3 was modeled on results from RA analyses, it is possible that other diseases may exhibit similarly strong associations that persist over a large number of SNPs. This may be especially true when causal variants are multi-allelic, such as the relationship between Kringle repeat number in the apolipoprotein A gene and serum Lp(a) [5].

In summary, we have demonstrated that FDR, as implemented in the program QVALUE, appears appropriate for genome-wide association studies where the majority of tests will not be significant. However, caution should be employed when using it for fine mapping because these regions may contain a large number of highly significant associations. This biased distribution appears to poorly affect FDR performance. In circumstances in which a large number of significant results are identified, other approaches should be considered to control error rate such as the double trend test [6] or the use of haplotypes [7].

## List of abbreviations used

FDR: False Discovery Rate

FP: False positive

LD: Linkage Disequilibrium

RA: Rheumatoid Arthritis

SNP: Single Nucleotide Polymorphism

TP: True positive

## Competing interests

The author(s) declare that they have no competing interests.
